# P-1736. Examining the Impact of Eliminating a Transitions of Care Pharmacist Program on Antimicrobial Use

**DOI:** 10.1093/ofid/ofae631.1899

**Published:** 2025-01-29

**Authors:** Mary Godsey, Angela Duenn, Curtis D Collins

**Affiliations:** Trinity Health Ann Arbor, Ann Arbor, Michigan; Trinity Health Ann Arbor, Ann Arbor, Michigan; Trinity Health Ann Arbor, Ann Arbor, Michigan

## Abstract

**Background:**

Antimicrobial stewardship interventions during transitions of care (TOC) have increased guideline concordant prescribing at hospital discharge. A TOC pharmacy program targeting discharging patients at the high risk for mortality was established at our community teaching hospital in 2016. A previous program analysis reported 14.4% of all pharmacist interventions made involved antimicrobials. The TOC pharmacy program was eliminated in 2022. The purpose of this study was to assess the impact of program elimination on appropriate and optimal antimicrobial prescribing.

**Methods:**

This retrospective, single center, pre-post cohort study investigated the impact of the elimination of the TOC program on appropriate and optimal antimicrobial prescribing. Adult inpatients with the highest risk of 30-day mortality receiving an antimicrobial at discharge between September - November 2021 (TOC pharmacist program cohort) and September - November 2022 (post-TOC cohort) were included. Appropriate antimicrobial use was defined as prescribing of indication-based effective and safe therapy. Optimal prescribing was defined as adherence to institutional guideline-based antimicrobial stewardship recommendations.

**Results:**

Six-hundred twenty-six patients were included with 313 patients in each cohort. Baseline characteristics between cohorts were generally similar with significant differences in the number of patients treated for community-acquired pneumonia (20.4% vs. 28.4%; *P* = 0.026) and bacteremias (11.5% vs. 5.1%; *P* = 0.004). There were no differences in appropriate (90.4% vs. 90.1%; *P* = 0.893), or optimal prescribing (80.8% vs. 78%; *P* = 0.374) between cohorts. There were no differences in incidence of non-optimal antimicrobial dosing (6.4% vs. 5.8%; *P* = 0.738), or non-optimal antimicrobial selection (1.3% vs. 2.9%; *P* = 0.161); however, the incidence of non-optimal duration decreased in the post-TOC cohort (22% vs. 15%; *P* = 0.024).

**Conclusion:**

The elimination of the TOC pharmacy program did not impact appropriate or optimal antimicrobial prescribing. Relatively high appropriate and optimal prescribing were noted in both cohorts and additional ongoing stewardship interventions may have confounded results.

**Disclosures:**

**All Authors**: No reported disclosures

